# DYT1 Dystonia Patient-Derived Fibroblasts Have Increased Deformability and Susceptibility to Damage by Mechanical Forces

**DOI:** 10.3389/fcell.2019.00103

**Published:** 2019-06-26

**Authors:** Navjot Kaur Gill, Chau Ly, Paul H. Kim, Cosmo A. Saunders, Loren G. Fong, Stephen G. Young, G. W. Gant Luxton, Amy C. Rowat

**Affiliations:** ^1^Department of Integrative Biology and Physiology, University of California, Los Angeles, Los Angeles, CA, United States; ^2^Department of Bioengineering, University of California, Los Angeles, Los Angeles, CA, United States; ^3^Department of Medicine, University of California, Los Angeles, Los Angeles, CA, United States; ^4^Department of Genetics, Cell Biology, and Development, University of Minnesota, Minneapolis, MN, United States; ^5^Department of Human Genetics, University of California, Los Angeles, Los Angeles, CA, United States; ^6^Molecular Biology Institute, University of California, Los Angeles, Los Angeles, CA, United States; ^7^Department of Biomedical Engineering, University of Minnesota, Minneapolis, MN, United States

**Keywords:** torsinA, LINC complex, mechanotype, cell mechanical properties, nuclear envelope, lamins

## Abstract

DYT1 dystonia is a neurological movement disorder that is caused by a loss-of-function mutation in the *DYT1*/*TOR1A* gene, which encodes torsinA, a conserved luminal ATPases-associated with various cellular activities (AAA+) protein. TorsinA is required for the assembly of functional linker of nucleoskeleton and cytoskeleton (LINC) complexes, and consequently the mechanical integration of the nucleus and the cytoskeleton. Despite the potential implications of altered mechanobiology in dystonia pathogenesis, the role of torsinA in regulating cellular mechanical phenotype, or mechanotype, in DYT1 dystonia remains unknown. Here, we define the deformability of mouse fibroblasts lacking functional torsinA as well as human fibroblasts isolated from DYT1 dystonia patients. We find that the deletion of torsinA or the expression of torsinA containing the DYT1 dystonia-causing ΔE302/303 (ΔE) mutation results in more deformable cells. We observe a similar increased deformability of mouse fibroblasts that lack lamina-associated polypeptide 1 (LAP1), which interacts with and stimulates the ATPase activity of torsinA *in vitro*, as well as with the absence of the LINC complex proteins, Sad1/UNC-84 1 (SUN1) and SUN2, lamin A/C, or lamin B1. Consistent with these findings, we also determine that DYT1 dystonia patient-derived fibroblasts are more compliant than fibroblasts isolated from unafflicted individuals. DYT1 dystonia patient-derived fibroblasts also exhibit increased nuclear strain and decreased viability following mechanical stretch. Taken together, our results establish the foundation for future mechanistic studies of the role of cellular mechanotype and LINC-dependent nuclear-cytoskeletal coupling in regulating cell survival following exposure to mechanical stresses.

## Introduction

Dystonia is a “hyperkinetic” neurological movement disorder, which is the third most common movement disorder worldwide behind essential tremor and Parkinson’s disease (Fahn, 1988; Geyer and Bressman, 2006; Defazio et al., 2007). Dystonia is characterized by involuntary sustained or intermittent muscle contractions resulting in abnormal repetitive movements and/or postures (Fahn, 1988; Albanese et al., 2013). While multiple treatment options are available for managing dystonia—such as botulinum toxin injection, oral medications, and deep brain stimulation—no curative therapies exist (Albanese et al., 2019). If we could fully define the mechanisms of dystonia pathogenesis, this would enable the development of effective targeted treatment strategies for dystonia patients.

Dystonia can be acquired as a result of traumatic brain injury, central nervous system infection, or environmental toxins (Albanese et al., 2013, 2019). This neurological disorder can also be inherited: the most prevalent and severe inherited dystonia (Weisheit et al., 2018), DYT1 dystonia, is caused by a loss-of-function mutation in the *DYT1*/*TOR1A* gene that deletes a single glutamic acid residue (ΔE302/303, or ΔE) from the encoded torsinA protein (Ozelius et al., 1997). TorsinA is an AAA+ protein, which resides within the lumen of the endoplasmic reticulum lumen and the contiguous perinuclear space of the nuclear envelope (Goodchild and Dauer, 2004; Naismith et al., 2004). AAA+ proteins typically function as ATP-dependent molecular chaperones that structurally remodel their protein substrates (Hanson and Whiteheart, 2005). While the substrate(s) remodeled by torsinA are unknown, torsinA is thought to function within the nuclear envelope where its ATPase activity is stimulated by its membrane-spanning co-factors: lamina-associated polypeptide 1 (LAP1) and luminal domain-like LAP1 (LULL1) (Laudermilch et al., 2016). While the ΔE mutation impairs the ability of torsinA to interact with or be stimulated by either LAP1 or LULL1 (Naismith et al., 2009; Zhao et al., 2013), a mechanistic understanding of how the ΔE mutation drives DYT1 dystonia pathogenesis at the cellular level remains unclear.

We recently identified torsinA and LAP1 as mediators of the assembly of functional linker of nucleoskeleton and cytoskeleton (LINC complexes) (Saunders and Luxton, 2016; Saunders et al., 2017), which are evolutionarily conserved nuclear envelope-spanning molecular bridges that mechanically integrate the nucleus and the cytoskeleton (Ansardamavandi et al., 2016). LINC complexes are composed of the outer nuclear membrane nuclear envelope spectrin repeat (nesprin) proteins and the inner nuclear membrane Sad1/UNC-84 (SUN) proteins. Nesprins interact with the cytoskeleton in the cytoplasm and SUN proteins in the perinuclear space, whereas SUN proteins interact with A-type lamins and chromatin-binding proteins in the nucleoplasm (Crisp et al., 2006; Wilson and Berk, 2010; Chang et al., 2015b). Our previous work demonstrated that torsinA and LAP1 are required for the assembly of transmembrane actin– associated nuclear (TAN) lines (Saunders et al., 2017), which are linear arrays of LINC complexes composed of the actin-binding nesprin-2Giant (nesprin-2G) and SUN2 that harness the forces generated by the retrograde flow of perinuclear actin cables to move the nucleus toward the rear of migrating fibroblasts and myoblasts; this is required for efficient directional migration (Luxton et al., 2010, 2011; Chang et al., 2015a). Consistent with these findings, DYT1 dystonia patient-derived fibroblasts and fibroblasts isolated from mouse models of DYT1 dystonia exhibit reduced motility *in vitro* (Nery et al., 2008, 2014). Moreover, the migration of torsinA-null neurons in the dorsal forebrain of torsinA-null mouse embryos show impaired migration *in vivo* (McCarthy et al., 2012). Since intracellular force generation is critical for cell motility, and regulated by shared mediators of mechanotype (Rodriguez et al., 2003; Herrmann et al., 2007; Dittmer and Misteli, 2011; Chung et al., 2013; Chang et al., 2015b; Xavier et al., 2016; Fritz-Laylin et al., 2017), these results suggest that DYT1 dystonia may be characterized by defective mechanobiology.

Here, we test the hypothesis that torsinA regulates cellular mechanical phenotype, or mechanotype, which describes how cells deform in response to mechanical stresses. Cellular mechanotype is critical for the process of mechanotransduction, whereby cells translate mechanical stimuli from their environment into biochemical signals and altered gene expression (Franze et al., 2013). The ability of cells to withstand physical forces is also crucial for their survival (Hsieh and Nguyen, 2005). For example, the external stresses of traumatic brain injury result in cell death (Raghupathi, 2004; Stoica and Faden, 2010; Hiebert et al., 2015; Ganos et al., 2016). Damage to cells can likewise occur during their migration through narrow constrictions, including nuclear rupture, DNA damage, and cell death (Harada et al., 2014; Denais et al., 2016; Raab et al., 2016; Irianto et al., 2017).

The damaging effects of such large cellular deformations depend on levels of A-type nuclear lamins, which are critical regulators of nuclear and cellular mechanotype (Lammerding et al., 2004; Swift et al., 2013; Stephens et al., 2017). The depletion of other proteins that associate with nuclear lamins, such as the inner nuclear membrane protein emerin, similarly result in reduced mechanical stability of the nuclear envelope (Rowat et al., 2006; Reis-Sobreiro et al., 2018) as well as increased nuclear strain following mechanical stretch (Lammerding et al., 2005). The nuclear lamina also interacts with chromatin, which can further contribute to the mechanical properties of the nucleus (Pajerowski et al., 2007; Chalut et al., 2012; Schreiner et al., 2015; Stephens et al., 2017). In addition, nuclear lamins associate with the LINC complex, which mediates the transmission of physical forces generated by the cytoskeleton across the nuclear envelope and into the nucleoplasm (Stewart-Hutchinson et al., 2008; Lombardi et al., 2011; Spagnol and Dahl, 2014). Given that torsinA is required for the assembly of both actin- and intermediate filament-binding LINC complexes in fibroblasts (Hewett et al., 2006; Nery et al., 2008, 2014; Vander Heyden et al., 2009; Saunders et al., 2017), we speculated that DYT1 dystonia patient-derived fibroblasts may exhibit altered mechanotype. We chose to study the effect of the loss of torsinA function on the mechanotype of fibroblasts for three main reasons: (1) fibroblasts have been used to successfully model human neurological disorders, including dystonia (Connolly, 1998; Hewett et al., 2006; Auburger et al., 2012; Burbulla and Kruger, 2012; Wray et al., 2012; Nery et al., 2014); (2) the molecular mediators of mechanotype are typically conserved across cell types (Rodriguez et al., 2003; Herrmann et al., 2007; Dittmer and Misteli, 2011; Chung et al., 2013; Chang et al., 2015b; Xavier et al., 2016; Fritz-Laylin et al., 2017), thus findings in fibroblasts may be extended to neurons; and (3) dermal fibroblasts can be easily isolated from DYT1 dystonia patients (Nery et al., 2014).

Here, we show that the deformability of human and mouse fibroblasts is altered due to the expression of torsinA containing the DYT1 dystonia-causing ΔE mutation (torsinA^Δ*E*^), the absence of torsinA, the deletion of LAP1, or the disruption of functional LINC complexes. We find that fibroblasts isolated from DYT1 dystonia patients are more deformable than normal human fibroblasts. Interestingly we also observe that DYT1 dystonia patient-derived fibroblasts exhibit nuclei with greater strain and decreased viability following mechanical stretching of their substrates. Collectively these results establish the roles of functional torsinA and LINC complexes in regulating cellular deformability. Our findings should guide future studies to better understand the pathophysiology of diseases ranging from dystonia to cancer, which are associated with mutations in the genes that encode torsin, LAP1 and/or LINC complex proteins (Luxton and Starr, 2014; Sewry and Goebel, 2014; Rebelo et al., 2015; Ghaoui et al., 2016; Janin et al., 2017; Kariminejad et al., 2017; Reichert et al., 2017).

## Materials and Methods

### DNA Constructs

The retroviral packaging cDNA constructs that were used to generate the NIH3T3 fibroblast cell lines that stably express EGFP-tagged torsinA constructs were created as follows: PCR was used to amplify the cDNA sequences encoding wild type (WT) or mutant versions of torsinA containing EGFP inserted after its signal sequence (SS) using the previously described SS-EGFP-torsinAWT, SS-EGFP-torsinAE171Q, or SS-EGFP-torsinAΔE constructs (Goodchild and Dauer, 2004; Saunders et al., 2017) as templates and the primers SS-EGFP-F (5′-GGGCGCCTCGAGATGAAGCTGGGCCGGG-3′) and SS-EGFP-torsinA-R (5′-GCGCCCGAATTCTCAATCATCGTAGTAATAATCTAACTTGGTG-3′), which contain 5′*XhoI* and *EcoRI* restriction enzyme (RE) cut sites, respectively. Each PCR product was purified and digested alongside the retroviral packaging vector pLPCX (Takara Bio USA, Inc., Mountain View, CA) with *XhoI* and *EcoRI*. The digested PCR products and pLPCX vectors were then gel purified, ligated together, and confirmed by sequencing performed at the University of Minnesota Genomics Center. Phusion DNA polymerase, REs, and T4 DNA ligase were purchased from New England Biolabs (NEB, Ipswich, MA). The Wizard SV Gel and PCR Clean-Up System used to purify the PCR products and digested DNA was purchased from Promega. The GeneJet Plasmid Midiprep Kit, which was used to purify each construct, was purchased from ThermoFisher Scientific (Waltham, MA, United States).

### Cells

Parental NIH3T3 fibroblasts were cultured in L-glutamine-, glucose-, and sodium pyruvate-containing Dulbecco’s modified Eagle’s media (DMEM) (Thermo Fisher Scientific, Waltham, MA, United States) supplemented with 10% bovine calf serum (BCS) (Gemini Bio-Products, West Sacramento, CA, United States). NIH3T3 fibroblasts stably expressing WT, or mutant (E171Q or ΔE) versions of torsinA were created as follows. The pLPCX vectors encoding each SS-EGFP-tagged torsinA construct were separately transfected along with the pVSV-G (Takara Bio USA, Inc.) construct, which encodes the vesicular stomatitis virus G envelope protein into the gp293 retroviral packaging cell line (Takara Bio USA, Inc.). The subsequent isolation of the pseudotyped retroviral particles produced by the gp293 cells was performed as recommended by the manufacturer. NIH3T3 fibroblasts were transduced with purified pseudotyped retroviruses and selected with 2 μg/mL puromycin (Thermo Fisher Scientific). Individual clones of the resultant cell lines were isolated using limiting dilution and maintained with 2 μg/mL of puromycin.

The *Tor1a*^+/+^, *Tor1a*^−/−^, *Tor1aip1*^+/+^, *Tor1aip1*^−/−^, *LMNA^+/+^*, *LMNA*^−/−^, *LMNB1*^+/+^, and *LMNB1*^−/−^ mouse embryonic fibroblasts (MEFs) used in this study were previously described (Kim et al., 2010; Jung et al., 2014; Saunders et al., 2017). The *Tor1a*^+/Δ*E*^ and *Tor1a*^Δ*E*/Δ*E*^ MEFs used here were a kind gift from Dr. William T. Dauer, who isolated them from his previously described ΔE knock-in mice (Goodchild et al., 2005). The SUN1/2^+/+^, SUN1/2^−/−^, and SUN2^−/−^ MEFs used here were a kind gift from Dr. Brian Burke, who isolated them from previously described mice (Lei et al., 2009). All the MEFs used in this study were grown in DMEM supplemented with 15% BCS and 1% penicillin, and streptomycin. Human fibroblasts (GM00023, GM00024, GM02912, GM03211, GM03221, and GM02304) were purchased from the Coriell Institute and cultured following vendor’s instructions (Camden, NJ, United States): GM00023, GM03211, GM03221, and GM02304 were grown in DMEM containing 15% fetal bovine serum (FBS) (Gemini Bio-Products, West Sacramento, CA, United States). GM00024 were grown in DMEM supplemented with 10% FBS. GM02912 were grown in 20% FBS-containing Ham’s F12 media supplemented with 2 mM L-glutamine (Sigma-Aldrich, St. Louis, MO, United States). All cells were grown at 37°C with 5% CO_2_.

### Parallel Microfiltration (PMF)

Prior to filtration measurements, cells were washed with 1× DNase-, RNase- and Protease-free phosphate-buffered saline purchased from Mediatech (Manassas, VA, United States), treated with trypsin (VWR, Visalia, CA, United States), and resuspended in fresh medium to a density of 0.5 × 10^6^ cells/mL. Cell suspensions were maintained for 30 min after harvesting to enable cells to round following their detachment from the substrate; the rounding of detached cells typically occurs over timescales of ∼min (Shamik and Kumar, 2009). Prior to each filtration measurement, cell suspensions were passed through a 35 μm cell strainer (BD Falcon, San Jose, CA, United States). Next, 350 μL of each cell suspension was loaded into each well of a 96-well loading plate (Greiner BioOne, Monroe, NC, United States). The number and size distribution of cells in each well were quantified using an automated cell counter (TC20, BioRad Laboratories, Hercules, CA, United States). Finally, a defined magnitude of air pressure which was monitored using a 0–100 kPa pressure gauge (Noshok Inc., Berea, OH, United States), was applied to the 96-well plate outfitted with a custom pressure chamber for 40 or 50 s (Qi et al., 2015; Gill et al., 2017). To quantify retention volumes following filtration, we measured the absorbance at 560 nm of the phenol red-containing cell medium using a plate reader (Infinite M1000, Tecan Group Ltd., Männedorf, Switzerland).

### Quantitative Deformability Cytometry (q-DC)

Standard soft lithography methods were used to fabricate the microfluidic devices for q-DC experiments. Briefly, a 10:1 w/w base to crosslinker ratio of polydimethylsiloxane (PDMS) was poured onto a previously described master wafer (Nyberg et al., 2016). The device was subsequently bonded to a No. 1.5 glass coverslip (Thermo Fisher Scientific) using plasma treatment (Plasma Etch, Carson City, NV, United States). Within 24 h of device fabrication, suspensions of 2 × 10^6^ cells/mL were driven through constrictions of 9 μm (width) × 10 μm (height) by applying 55 kPa of air pressure across the device. We captured images of cell shape during transit through the 9 μm gaps on the millisecond timescale using a CMOS camera with a capture rate of 1600 frames/s (Vision Research, Wayne, NJ, United States) mounted on an inverted Axiovert microscope (Zeiss, Oberkochen, Germany) equipped with a korr Ph2 20x/0.4NA LD Achroplan objective (Zeiss) and light source (Osram Halogen Optic Lamp 100 W, 12 V). We used custom MATLAB (MathWorks, Natick, MA, United States) code^1^ to analyze the time-dependent shape and position changes of individual cells (Nyberg et al., 2016). To determine the mechanical stresses applied to individual cells, we used agarose calibration particles that were fabricated using oil-in-water emulsions as previously described (Nyberg et al., 2017). Stress-strain curves were obtained for single cells and a power-law rheology model was subsequently fitted to the data to compute cellular elastic modulus.

### Epifluorescence Microscopy

To image cell and nuclear morphology, cells grown on No. 1.5 coverslips were labeled with 5 μM Calcein-AM and 0.2 μg/mL Hoechst 33342 (ThermoFisher Scientific). For cell viability measurements, cells were stained with 50 μg/mL propidium iodide (Thermo Fisher Scientific). Images of fluorescently labeled cells were acquired using a Zeiss Axio Observer A.1 microscope equipped with a 10x/0.3 NA EC Plan-Neofluar Ph1 M27 objective, a 20×/0.8 NA Plan-Apochromat M27 objective, a HBO 103W/2 mercury vapor short-arc lamp light source, a BP 470/20 excitation filter, a BP 505–530 emission filter, and a FT 495 beam splitter. ImageJ (Bethesda, MA, United States) was used to quantify cell and nuclear size and shape parameters from the acquired images.

### Cell Stretching

To subject cells to external mechanical stresses, we used a custom-built cell stretching apparatus (Kim et al., 2018). We prepared elastic PDMS membranes as previously described (Kim et al., 2018). Cells were resuspended in tissue culture media at a concentration of 5 × 10^5^ cells/mL and then added to individual PDMS strips, which were incubated for 24 h at 37°C with 5% CO_2_. To quantify nuclear strain, the membranes with cells adhered were stretched by 2 mm (5% of the total length of the membrane) while submerged in cell culture media for 5 min prior to imaging. To determine the effects of repetitive stretch on cell adhesion and viability, membranes were stretched by 2 mm at 0.5 Hz for 24 h at 37°C. After 24 h, membrane-adhered cells were stained with fluorescent dyes and imaged as described above. To quantify the number of cells attached to the membranes after stretching, we prepared lysates of the adherent cells using a solution of 0.1 N NaOH (Sigma-Aldrich) and measured the total protein content of the lysates using the detergent compatible (DC) protein assay kit (BioRad Laboratories).

### Statistical Methods

To determine the statistical significance of data that exhibited non-parametric distributions (*TT*, *E*_a_, and cell size) we used the Mann–Whitney *U*-test. To determine the statistical significance of data with unequal variances (nuclear size, shape, and strain) we used the Welch’s *t*-test. We used Cohen’s *d* test to determine the effect size for differences observed in nuclear strain. To determine the statistical differences in the variability of nuclear shape across samples, we used Levene’s test. All other results were analyzed using the Student’s *t*-test method to determine statistical significance.

## Results

### TorsinA and LAP1 Contribute to Fibroblast Deformability

**FIGURE 1:**
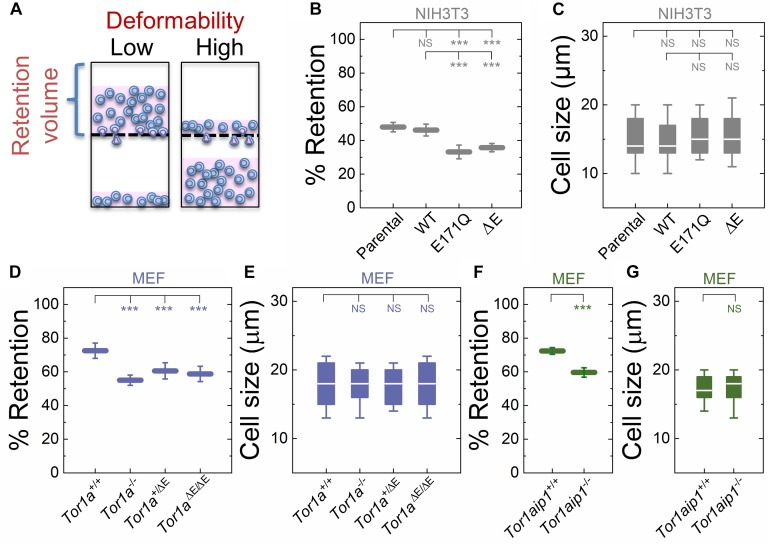
Fibroblasts lacking functional torsinA or LAP1 have increased deformability relative to controls. **(A)** Schematic illustration of PMF. Less deformable cells tend to occlude pores, thereby blocking fluid flow and resulting in an increased volume of fluid that is retained above the membrane. The retained fluid volume relative to the initial loaded volume is % retention. **(B)** PMF measurements of NIH3T3 fibroblasts expressing the indicated SS-EGFP-tagged torsinA constructs. **(D,F)** PMF measurements of the indicated MEF lines. PMF conditions: 10 μm pore size, 2.1 kPa for 50 s. Each data point represents the mean ± standard deviation (SD). Statistical significance was determined using Student’s *t*-test. **(C,E,G)** Cell size data. Boxplots show 25 and 75th percentiles; line shows median; and whiskers denote 10 and 90th percentiles. All data were obtained from three independent experiments. Statistical significance was determined using Mann-Whitney *U*-test. ^∗∗∗^*p* < 0.001; not significant (NS) *p* > 0.05.

To begin to determine if DYT1 dystonia is associated with altered cellular deformability, we performed our previously described PMF assay (Qi et al., 2015; Gill et al., 2017) on NIH3T3 fibroblasts with impaired torsinA function. In PMF, cell suspensions are filtered through porous membranes on the timescale of seconds by applying a defined magnitude of air pressure. Cells that occlude the micron-scale pores due to their stiffness and/or size block the fluid flow, reducing filtrate volume, and increasing the volume of fluid that is retained in the top well, which we report as % retention (Figure 1A). While the PMF setup is similar to a transwell migration assay where cells actively migrate through confined spaces, the timescale for cell migration is ∼hours (Justus et al., 2014; Paul et al., 2017), whereas the timescale of filtration measurements is ∼seconds (Qi et al., 2015); therefore PMF provides a measurement of the ability of cells to passively deform through micron-scale pores (Qi et al., 2015; Kim et al., 2016; Nyberg et al., 2018; Reis-Sobreiro et al., 2018).

To manipulate torsinA function, we generated lentivirus-transduced NIH3T3 fibroblasts that stably express previously described cDNA constructs encoding WT or mutant (E171Q or ΔE) torsinA (Saunders et al., 2017). The E171Q mutation inactivates the Walker B site in the AAA+ domain of torsinA, which prevents torsinA from hydrolyzing ATP. Since neither SS-EGFP-torsinA^*E*171*Q*^ nor SS-EGFP-torsinA^Δ*E*^ were able to rescue the rearward nuclear positioning and centrosome orientation defects observed in MEFs isolated from torsinA-knockout (*Tor1a*^−/−^) mice (Goodchild et al., 2005; Saunders et al., 2017), we rationalized that these constructs would act as dominant negative inhibitors of torsinA function in NIH3T3 fibroblasts. We found that NIH3T3 fibroblasts expressing either SS-EGFP-torsinA^*E*171*Q*^ or SS-EGFP-torsinA^Δ*E*^ exhibited significantly lower % retention than parental non-transduced or SS-EGFP-torsinA^*WT*^ transduced NIH3T3 fibroblasts (Figure 1B). While cell size can impact filtration (Qi et al., 2015; Nyberg et al., 2017), we observed no significant differences in size distributions across these cell lines (Figure 1C), suggesting that their altered filtration is due to differences in cellular deformability. These data indicate that torsinA regulates the deformability of NIH3T3 fibroblasts.

To further investigate the relationship between torsinA function and cellular deformability, we next performed PMF experiments on previously characterized *Tor1a*^+/+^ and *Tor1a*^−/−^ MEFs (Saunders et al., 2017). We found that % retention of the *Tor1a*^+/+^ MEFs was significantly larger than % retention of the *Tor1a*^−/−^ MEFs (Figure 1D). Consistent with these findings, we observed that MEFs isolated from heterozygous (*Tor1a*^+/Δ*E*^) or homozygous (*Tor1a*^Δ*E*/Δ*E*^) ΔE-knock-in mice (Goodchild et al., 2005) had a significantly lower % retention than *Tor1a*^+/+^ MEFs (Figure 1D). Moreover, % retention measured for MEFs derived from LAP1-knockout (*Tor1aip1*^−/−^) mice was also significantly lower than % retention measured for control *Tor1aip1*^+/+^ MEFs (Figure 1F). We confirmed that these observed changes in % retention were not due to significant differences in cell size distributions (Figure 1E,G). Because the interaction between torsinA and the luminal domain of LAP1 stimulates its ATPase activity *in vitro* (Zhao et al., 2013) and the ΔE mutation impairs the ability of torsinA to interact with LAP1 (Naismith et al., 2009), these results suggest that the interaction between torsinA and LAP1 may contribute to fibroblast deformability. In addition, LAP1 is critical for nuclear envelope structure (Kim et al., 2010; Santos et al., 2015) and interacts with nuclear lamins (Foisner and Gerace, 1993; Serrano et al., 2016), which are major determinants of cellular mechanotype (Houben et al., 2007). Thus, the impaired interaction between torsinA and LAP1 caused by the ΔE mutation may also contribute to the increased deformability of *Tor1aip1*^−/−^ MEFs.

### The LINC Complex and Nuclear Lamins Mediate Cellular Deformability

**FIGURE 2:**
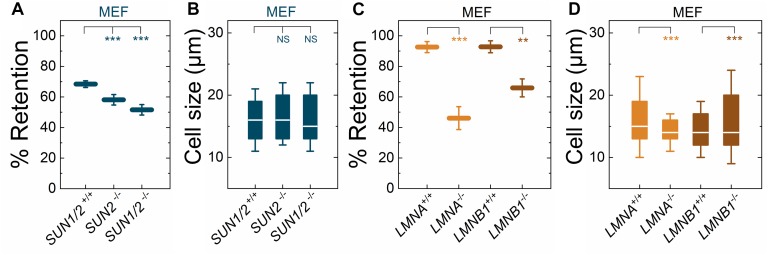
Fibroblasts lacking LINC complexes (SUN1/2, SUN2), A-type lamins (LMNA), or lamin B1 (LMNB1) have increased deformability relative to controls. PMF measurements of the indicated MEF lines. **(A)** PMF conditions: 10 μm pore membrane, 2.1 kPa for 50 s. **(C)** PMF conditions: 10 μm pore membrane, 2.1 kPa for 40 s. Each data point represents the mean ± SD. Statistical significance was determined using Student’s *t*-test. **(B,D)** Cell size data. Boxplots show 25 and 75th percentiles; line shows median; whiskers denote 10 and 90th percentiles. Statistical significance was determined using Mann–Whitney *U*-test. All data were obtained from three independent experiments. ^∗∗∗^*p* < 0.001; ^∗∗^*p* < 0.01; not significant (NS) *p* > 0.05.

We previously showed that torsinA and LAP1 are both required for nuclear-cytoskeletal coupling through SUN2-containing LINC complexes (Saunders et al., 2017). Thus, we next asked whether or not MEFs isolated from SUN2-knockout (*SUN2*^−/−^) mice (Lei et al., 2009) exhibited similar decreased filtration as the *Tor1a*^−/−^, *Tor1a*^+/Δ*E*^, *Tor1a*^Δ*E*/Δ*E*^, and *Tor1aip1*^−/−^ MEFs. We found that *SUN2*^−/−^ MEFs had reduced % retention relative to control (*SUN1/2*^+/+^) MEFs (Figure 2A). Since torsinA has also been proposed to interact with and regulate SUN1-containing LINC complexes (Jungwirth et al., 2011; Weisheit et al., 2018; Chalfant et al., 2019), we also performed PMF on MEFs derived from SUN1/2-double knockout (*SUN1/2*^−/−^) mice (Lei et al., 2009) and found that they had reduced % retention relative to both *SUN2*^−/−^ and *SUN1/2*^+/+^ MEFs (Figure 2A). These changes in % retention were not due to significant differences in cell size distributions (Figure 2B).

To determine how perturbations of the LINC complex compare to depletion of an established regulator of nuclear and cellular mechanotype, we next investigated the effects of A-type lamins (Lammerding et al., 2004; Swift et al., 2013); these nuclear-specific intermediate filament proteins directly interact with LAP1 (Foisner and Gerace, 1993; Serrano et al., 2016) as well as SUN1 and SUN2 (Chang et al., 2015a). We found that MEFs isolated from lamin A/C-knock-out (*LMNA*^−/−^) mice exhibited reduced % retention relative to MEFs isolated from control mice (*LMNA*^+/+^) (Figure 2C). The increased deformability of the *LMNA*^−/−^ MEFs that we observed is consistent with previous reports from our laboratory and others, which show that A-type lamins determine the ability of cells to deform through micron-scale pores, both during passive deformation driven by applied pressure (timescale ∼ seconds) and active migration (timescale ∼ hours) (Rowat et al., 2013; Harada et al., 2014). In addition to A-type nuclear lamins, many cells types express the B-type nuclear lamins, lamin B1 and lamin B2 (Dittmer and Misteli, 2011; Reddy and Comai, 2016). Lamin B1 interacts with SUN1 (Nishioka et al., 2016) and LAP1 (Maison et al., 1997) and is required for proper nuclear-cytoskeletal coupling (Ji et al., 2007). We found that MEFs isolated from lamin-B1-knockout mice (*LMNB1*^−/−^) (Vergnes et al., 2004) had reduced % retention compared to MEFs isolated from control mice (*LMNB1*^+/+^) (Figure 2C), suggesting that lamin B1 also contributes to cellular deformability. These findings are in agreement with previous findings that identify lamin B1 as a determinant of nuclear shape and stiffness (Coffinier et al., 2011; Ferrera et al., 2014). While we observed differences in cell size distributions between *LMNA*^−/−^ and *LMNA*^+/+^ MEFs as well as between *LMNB1*^−/−^ and *LMNB1*^+/+^ MEFs, we did not observe that cells with larger median cell size had increased % retention (Figure 2C,D). Taken together, these results suggest that nuclear lamins, torsinA, LAP1, and LINC complexes are important mediators of cellular deformability. These results are consistent with a model where cells are more deformable when the mechanical integration of the nucleus and the cytoskeleton is perturbed.

### DYT1 Dystonia Patient-Derived Fibroblasts Are More Deformable Than Control Fibroblasts

**FIGURE 3:**
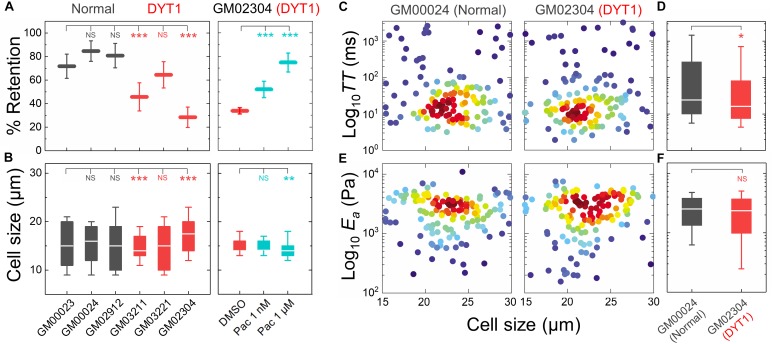
DYT1 dystonia patient-derived fibroblasts have increased deformability compared to controls. **(A)** PMF measurements of normal and DYT1 dystonia patient-derived fibroblast lines. Paclitaxel (Pac) treatment for 24 h prior to filtration. PMF conditions: 10 μm pore membrane, 1.4 kPa for 50 s. Each data point represents the mean ± SD. Statistical significance was determined using Student’s *t*-test. **(B)** Cell size data. Boxplots show 25 and 75th percentiles; line shows median; whiskers denote 10 and 90th percentiles. **(C,E)** Density scatter plots for *TT* and *E*_a_ measurements determined by q-DC. Each dot represents a single cell. *N* > 190 per sample. **(D,F)** *TT* and *E*_a_ measurement boxplots show 25 and 75th percentiles; line shows median; whiskers denote 10 and 90th percentiles. Statistical significance was determined using Student’s t-test. All data were obtained from three independent experiments. ^∗∗∗^*p* < 0.001; ^∗∗^*p* < 0.01; ^∗^*p* < 0.05; and not significant (NS) *p* > 0.05.

Having established that the DYT1 dystonia-causing ΔE mutation in torsinA makes cells more deformable and that torsinA, LINC complexes, and LINC complex-associated proteins are important determinants of cellular deformability, we next tested if fibroblasts isolated from DYT1 dystonia patients display defects in cellular mechanotype. We performed PMF on a panel of age-matched human fibroblasts isolated from normal individuals (GM00023, GM00024, and GM02912) and DYT1 dystonia patients (GM03211, GM03221, and GM02304). We found that DYT1 dystonia patient-derived fibroblasts had consistently lower % retention compared to fibroblasts isolated from unafflicted controls (Figure 3A). While there were some differences in cell size distributions across the DYT1 dystonia-derived fibroblast lines (Figure 3B), we consistently found that they exhibited lower % retention than the control fibroblasts. Notably the GM02304 line, which had the largest median cell size, exhibited the lowest % retention of all patient-derived fibroblasts (Figure 3A,B). To validate the effects of decreased cell deformability—or increased % retention—in our PMF assay, we determined the effects of paclitaxel treatment on the % retention of DYT1 dystonia patient-derived fibroblasts (Figure 3A,B). Previously, we identified the microtubule-stabilizing drug paclitaxel as a treatment that increases the % retention, or decreases cellular deformability, of human leukemia and ovarian cancer cells using PMF (Qi et al., 2015; Gill et al., 2019). Paclitaxel treatment recapitulates the decreased deformability that results from lamin A over-expression, as shown by the similar increased timescales required for cells to deform through narrow gaps (Rowat et al., 2013; Lautscham et al., 2015). Our findings indicate that stabilizing microtubules in DYT1 dystonia patient-derived fibroblasts with paclitaxel increases their % retention, consistent with a decreased whole cell deformability (Figure 3A); these findings contrast the increased cellular deformability we observe with perturbations of torsinA, LINC complex proteins, and nuclear lamins. Taken together, these findings suggest that fibroblasts isolated from DYT1 dystonia patients are more deformable than control fibroblasts, as assessed by their ability to deform through micron-scale pores on the timescale of seconds.

To validate the more compliant mechanotype of the DYT1 dystonia patient-derived fibroblasts (GM02304) versus control cells (GM00024), we used q-DC (Nyberg et al., 2017). q-DC enables single-cell measurements of transit time (*TT*), which is the time that it takes a cell to transit into the micron-scale constriction of a microfluidic device in response to applied pressure, and apparent elastic modulus (*E*_a_). We found that fibroblasts isolated from DYT1 dystonia patients had reduced median *TT* relative to control fibroblasts (median *TT*_GM02304_ = 16.2 ms versus *TT*_GM00024_ = 24.4 ms, *p* = 1.5 × 10^−2^) (Figure 3C,D). Since *TT* tends to be shorter for more compliant cells with reduced elastic modulus (Nyberg et al., 2017), these findings corroborate the increased deformability of DYT1 patient-derived fibroblasts that we observed using PMF. q-DC measurements can also be impacted by cell size, but we found no significant correlations of q-DC measurements with cell diameter (*d*) by linear regression analysis (Pearson’s r_GM02304_TT *vs.* d_ = 0.0, Pearson’s r_GM00024_TT *vs.* d_ = −0.1), suggesting that these observations of the altered DYT1 dystonia-derived fibroblast deformability do not depend on cell size. Using power law rheology to extract measurements of *E*_a_ (Nyberg et al., 2017), we found that DYT1 dystonia patient-derived fibroblasts have reduced median *E*_a_ compared to controls, although the reduction was not statistically significant (Figure 3E,F). Collectively, our PMF and q-DC measurements indicate that DYT1 dystonia patient-derived fibroblasts are more deformable than control fibroblasts.

### DYT1 Dystonia Patient-Derived Fibroblasts Display Altered Nuclear Morphology

Cellular and nuclear shape reflect a balance between cell-matrix adhesions, cellular force generation, mechanical stability of the cellular cortex and nuclear envelope, as well as nuclear-cytoskeletal connectivity (Dahl et al., 2004, 2008; Rowat et al., 2008; Murrell et al., 2015). Since the expression of torsinA^Δ*E*^ alters the mechanical integration of the nucleus and the cytoskeleton via the LINC complex (Nery et al., 2008; Jungwirth et al., 2011; Saunders et al., 2017), we hypothesized that fibroblasts isolated from DYT1 dystonia patients may have altered nuclear size and shape. To characterize these features, we performed quantitative image analysis of cells with fluorescently labeled cytoplasm (Calcein AM) and nuclei (Hoechst 33342) using epifluorescence microscopy. Since intracellular forces pulling on the nucleus in adhered cells can result in an increased nuclear area (Iyer et al., 2012), we first measured the projected area of nuclei in these cells, but found no significant differences between fibroblasts isolated from DYT1 dystonia patients (GM03211, GM03221, and GM02304) or controls (GM00023 and GM00024) (Supplementary Figure 1A). We also found no statistically significant differences between the cellular area of adhered DYT1 dystonia patient-derived and normal fibroblasts (Supplementary Figure 1B). Cell-to-nuclear size ratio was also similar across cell types, indicating that nuclear and cellular size scale similarly in DYT1 dystonia patient-derived and control fibroblasts (Supplementary Figure 1C).

**FIGURE 4:**
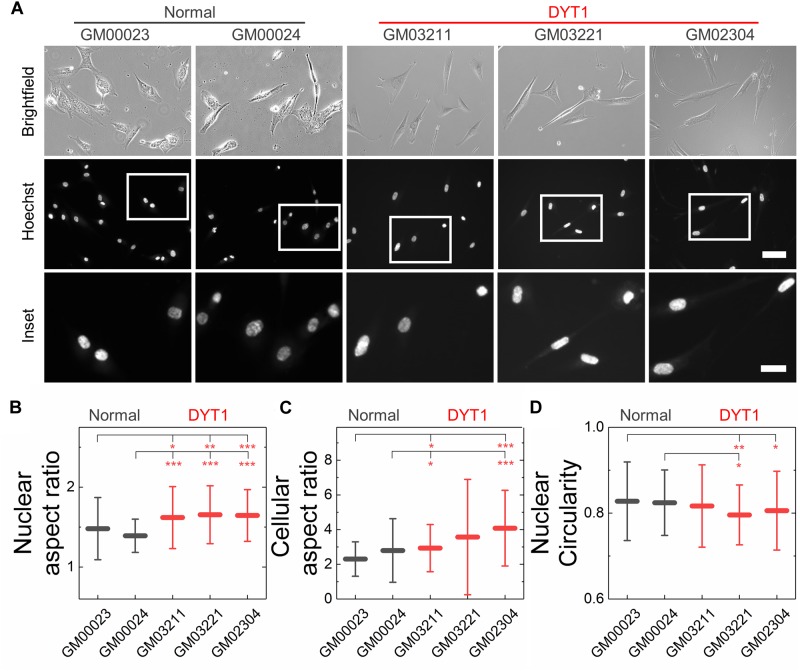
DYT1 dystonia patient-derived fibroblasts and nuclei are slightly more elongated relative to controls but do not exhibit marked differences in shape. **(A)** Representative brightfield and epifluorescence images of normal and DYT1 dystonia patient-derived fibroblasts and their nuclei. Nuclei were labeled with the fluorescent DNA dye, Hoeschst 33342. Scale, 20 μm. Inset: scale, 10 μm. Quantification of **(B)** nuclear aspect ratio, **(C)** cellular aspect ratio, and **(D)** nuclear circularity. Each data point represents the mean ± SD. Data were obtained from *N* > 30 cells across three independent experiments. Statistical significance was determined using the Welch’s *t*-test. ^∗∗∗^*p* < 0.001; ^∗∗^*p* < 0.01; ^∗^*p* < 0.05; and not significant (NS) *p* > 0.05 is not indicated on these plots for clarity.

We next investigated nuclear shape, which is impacted by cytoskeletal-generated forces as well as the inherent mechanical stability of the nuclear envelope in adhered cells (Rowat et al., 2008; Makhija et al., 2016). To quantify nuclear shape, we measured common metrics including aspect ratio and circularity *C*. We found that nuclei in fibroblasts isolated from DYT1 dystonia patients have a slightly larger aspect ratio than normal fibroblast nuclei, indicating that they were more elongated than nuclei in control fibroblasts (Figure 4A,B). Consistent with this observation, we also found DYT1 dystonia patient-derived fibroblasts have an increased cellular aspect ratio compared to normal control fibroblasts (Figure 4A,C). We further investigated nuclear circularity, *C*; this shape parameter is sensitive to irregular shapes that deviate from a circle, as

$$C=4\pi \frac{\text{Area}}{{\text{Perimeter}}^{2}}$$

with *C* = 1 for a perfect circle. However, we observed only minor differences in nuclear circularity between DYT1 dystonia patient-derived and normal fibroblasts (Figure 4D), consistent with the slightly elongated nuclear shapes that we observed in the fibroblasts isolated from DYT1 dystonia patients. Our findings that the DYT1 dystonia-causing ΔE mutation does not have a major impact on nuclear shape contrasts the known effects of reductions or mutations in A-type lamins (Rowat et al., 2013; Harada et al., 2014; Reis-Sobreiro et al., 2018), which are associated with nuclear blebbing, or lobulations, and tend to markedly reduce nuclear circularity (Funkhouser et al., 2013; Rowat et al., 2013; Reis-Sobreiro et al., 2018).

### DYT1 Dystonia Patient-Derived Fibroblasts Are More Susceptible to Damage Following Mechanical Stretch Than Control Fibroblasts

**FIGURE 5:**
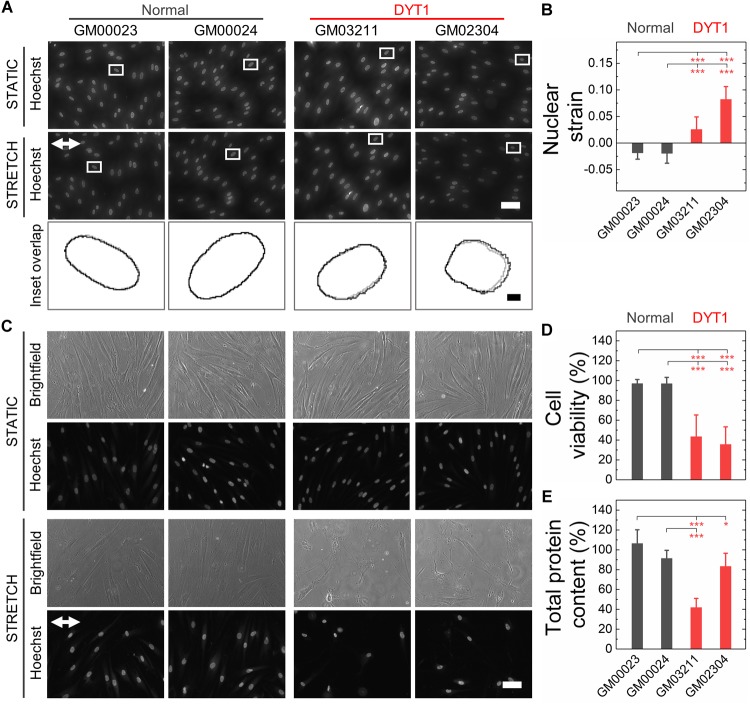
DYT1 dystonia patient-derived fibroblasts exhibit increased nuclear strain and are more susceptible to damage than controls upon mechanical stretch. **(A)** Representative images of normal and DYT1 dystonia patient-derived fibroblasts stained with Hoechst 33342. Nuclear morphology was examined after cells were exposed to mechanical stretch (5% strain) for 5 min. Images show cells under stretched (Stretch) or non-stretched (Static) conditions. White arrows denotes the direction of uniaxial stretch. Scale, 20 μm. The magnified inset shows overlapping outlines of nuclei from static (gray) and stretched (black) cells. Inset: scale, 1 μm. **(B)** Quantification of nuclear strain (change in nuclear area) due to cell stretching *N* > 15 cells. Statistical significance was determined using the Welch’s *t*-test. **(C)** Representative images of normal and DYT1 dystonia patient-derived fibroblasts after exposure to 24 h cyclical stretch (5% strain) and static conditions. Cells were stained with Hoechst 33342 to visualize nuclei by epifluorescence microscopy. Scale, 20 μm. **(D)** Cell viability of normal and DYT1 dystonia patient-derived fibroblasts after exposure to stretch for 24 h and static conditions. The viability data for stretched cells is normalized to static conditions for each cell line. *N* > 500 cells. **(E)** Quantification of the total protein content measured from biochemical lysates from cells that were adhered to the PDMS membranes after being exposed to cyclic stretch for 24 h. Total protein content of stretched cells was normalized to the total protein content of cells under static conditions for each cell line. Each data point represents the mean ± SD. Data were obtained from two independent experiments. Scale, 20 μm. Statistical significance was determined using Student’s *t*-test. ^∗∗∗^*p* < 0.001; ^∗^*p* < 0.05; and NS *p* > 0.05 is not indicated on these plots for clarity.

Nuclear lamins are critical for cell survival following exposure to physical forces, suggesting that the mechanical stability of the nucleus imparts protection from external mechanical stresses (Dahl et al., 2004; Denais et al., 2016; Raab et al., 2016; Irianto et al., 2017; Chen et al., 2018; Kim et al., 2018). Since fibroblasts isolated from DYT1 dystonia patients are more deformable than controls, we next tested the hypothesis that DYT1 dystonia patient-derived fibroblasts are more sensitive to damage caused by externally applied mechanical forces. We plated DYT1 dystonia patient-derived (GM03211 and GM02304) and control (GM00023 and GM00024) fibroblasts on an elastic collagen-coated PDMS membrane and subjected the resultant membrane with adhered cells to uniaxial mechanical stretch (5% strain). To quantify the magnitude of strain experienced by nuclei in these fibroblasts, we acquired images of cells under static and stretched conditions. We found that DYT1 dystonia patient-derived fibroblasts exhibited slightly larger changes in nuclear area (strain) relative to control fibroblasts in response to the same magnitude of strain applied to their substrate (Figure 5A,B). While the observed nuclear strain in GM02304 (*N* = 16) and GM03211 (*N* = 17) DYT1 dystonia patient-derived fibroblasts was only ∼9 and ∼3% larger than the control cells, these differences were nonetheless significant as determined by Cohen’s *d* test (*d* > 2). These observations of increased nuclear strain are consistent with the DYT1 dystonia patient-derived fibroblast nuclei being more deformable than nuclei in control fibroblasts. Despite the requirement of torsinA for nuclear-cytoskeletal coupling via the LINC complex in fibroblasts (Saunders et al., 2017), our findings suggest that nuclei in fibroblasts isolated from DYT1 dystonia patients are deforming more in response to external mechanical stresses than control fibroblast nuclei.

Since the mechanical stability of the nucleus is critical for cell survival following exposure to mechanical stresses (Harada et al., 2014; Denais et al., 2016; Raab et al., 2016; Irianto et al., 2017), we next determined the viability of DYT1 dystonia patient-derived (GM03211 and GM02304) and control (GM00023 and GM00024) fibroblasts after repeated cycles of stretch and relaxation at 0.5 Hz and 5% strain over 24 h. Visual inspection of fibroblasts in stretched versus static samples revealed major differences between fibroblasts isolated from DYT1 dystonia patients compared to control fibroblasts after 24 h. In contrast to the aligned morphologies of the control fibroblasts, which appeared similar in both stretched and static samples, the stretched DYT1 dystonia patient-derived fibroblasts were misaligned and exhibited irregular shapes (Figure 5C). While there was variability in nuclear shape across these cell lines in both static and stretched conditions, we confirmed that there were no statistically significant differences in the variance of nuclear shape across samples and independent biological replicates (Supplementary Table 1).

To evaluate the effect of mechanical stretching on the viability of the DYT1 dystonia patient-derived and control fibroblasts, we acquired images of cells stained with the established live/dead cell stain propidium iodide and used image analysis to quantify the number of dead cells that remained adhered to the substrate. While normal fibroblasts showed no significant cell death after stretching, we observed a marked 57–62% reduction in the viability of DYT1 dystonia patient-derived fibroblasts relative to static control fibroblasts, indicating their reduced survival following exposure to mechanical stresses (Figure 5D and Supplementary Figure 2). Since the response of cells to stretch depends on cell-substrate adhesions, we also assessed the number of cells that remained adhered to the PDMS substrate after stretching by quantifying the total protein content of cells lysed from the PDMS membrane. We found a significant ∼17–58% reduction in protein content for the stretched fibroblasts isolated from DYT1 dystonia patients as compared to static control fibroblasts, showing that there was significant detachment of DYT1 dystonia patient-derived fibroblasts from the substrate over the 24 h stretching period. By contrast, over 90% of the control fibroblasts were adhered to the membrane after 24 h (Figure 5E). The increased detachment of DYT1 dystonia patient-derived fibroblasts is consistent with a previous report of altered integrin-mediated adhesion in these cells (Hewett et al., 2006). Collectively, these findings indicate a striking difference in the response of DYT1 dystonia patient-derived fibroblasts to mechanical stretch.

## Discussion

Here, we show that DYT1 dystonia patient-derived fibroblasts are characterized by defective cellular mechanobiology: they have a more compliant mechanotype than fibroblasts isolated from non-afflicted individuals. We substantiate this finding by demonstrating that torsinA-null or torsinA^Δ*E*^-expressing MEFs are more deformable than wild-type control fibroblasts. Similarly, we show that MEFs lacking LAP1, SUN1, SUN2, lamin A, or lamin B1, are more deformable that wild-type controls. These findings are consistent with a model where torsinA, LINC complexes, and LINC complex-associated proteins each contribute to cellular deformability, which is ultimately determined by a network of interconnected cytoskeletal and nuclear proteins; consequently, the perturbation of any nodes in this network structure, or “mechanome”, can alter cellular mechanical properties (Rowat et al., 2006; Nakamura et al., 2009; Swift et al., 2013). TorsinA, LAP1, SUN1, SUN2, and lamin A/C are all critically important mediators of nuclear-cytoskeletal coupling in fibroblasts (Folker et al., 2011; Luxton et al., 2011; Saunders et al., 2017; Chang et al., 2019). Moreover, LAP1 directly interacts with and stimulates the ATPase activity of torsinA *in vitro* (Zhao et al., 2013) and the ΔE mutation impairs the ability of torsinA to interact with LAP1 (Naismith et al., 2009). LAP1 also interacts with nuclear lamins and the inner nuclear membrane protein emerin (Shin et al., 2014), which themselves directly interact with LINC complex proteins (Kim et al., 2015). Emerin was previously shown to be an important determinant of cellular mechanotype (Rowat et al., 2006; Chang et al., 2014; Reis-Sobreiro et al., 2018).

Our results also show that the expression of torsinA^Δ*E*^ is sufficient to impact cellular deformability: torsinA^Δ*E*^-expressing NIH3T3 fibroblasts, torsinA-null (*Tor1A*^−/−^) MEFs, heterozygous torsinA^Δ*E*^-knock-in (*Tor1a*^+/Δ*E*^) MEFs, homozygous torsinA^Δ*E*^-knock-in (*Tor1a*^Δ*E*/Δ*E*^) MEFs, and human fibroblasts isolated from DYT1 dystonia patients heterozygous for the ΔE mutation all exhibited increased filtration relative to control cells. Since AAA+ proteins typically function as homo-oligomeric molecular chaperones (Hanson and Whiteheart, 2005), the mere presence of the ΔE mutation may inhibit the torsinA homo-oligomer by preventing contact and/or communication between torsinA monomers, as suggested by previous reports that show torsinA^Δ*E*^ acts as a dominant negative inhibitor of torsinA function (Hewett et al., 2000; Kustedjo et al., 2000; Torres et al., 2004). However, it is important to note that the DYT1 dystonia genotype is heterozygous for the ΔE mutation and only 30–40% of individuals who possess this genotype develop dystonia (Ozelius et al., 1997). The low penetrance of the ΔE mutation is consistent with the fact that heterozygous torsinA^Δ*E*^-knock-in mice do not exhibit neuronal nuclear envelope blebbing nor perinatal lethality, as observed in torsinA-null or homozygous torsinA^Δ*E*^-knock-in mice (Goodchild et al., 2005). Thus, the ΔE mutation may not be a simple loss-of-function mutation, as previously suggested (Cookson and Clarimon, 2005). Future work will determine if the severity of change in filtration observed for a particular DYT1 dystonia patient-derived fibroblast line correlates with its performance in established assays for torsinA function, such as centrosome orientation (Saunders et al., 2017), directional cell migration (Nery et al., 2014), nuclear pore complex biogenesis (Laudermilch et al., 2016), and protein secretion (Hewett et al., 2006).

Here, we determined cellular deformability using the fluidic-based methods PMF and q-DC. While we have generally found good agreement between mechanotyping measurements performed using PMF, q-DC, and the well-established atomic force microscopy (AFM) (Kim et al., 2019), differences among deformability measurements can emerge due to differences in deformation time and length scales, as well as whether the cell was measured in a suspended or adhered state. In the PMF filtration-based measurement of cell deformability, cells in a suspended state flow through 10 μm pores over <1 min timescales. Since fibroblast diameter typically ranges from 14 to 18 μm, this method probes the ability of whole cells to passively deform on timescales faster than active cell migration. Major contributors to cell deformability in this assay are cortical and cytoplasmic components (e.g., actin), the nucleus (e.g., lamins), and nuclear-cytoskeletal connectivity (e.g., LINC complexes and their associated proteins), as we show here. Other flow-based methods, such as q-DC, similarly probe the ability of cells to deform through narrow gaps, albeit on faster deformation timescales of 10 to 100 ms. By contrast, AFM measures cell mechanical properties with much smaller deformations of nm to μm on timescales of sec to min. For AFM measurements, cells are typically adhered to a substrate; thus cortical stiffness as well as cell spreading and intracellular tension may also contribute to the measured cellular mechanotype. Future AFM studies will provide further insights into the mechanotype of adhered DYT1 dystonia patient-derived fibroblasts, including precise measurements of cortical stiffness relative to control fibroblasts. Using complementary mechanotyping methods to probe cellular deformability across varying time and length scales should also provide more detailed knowledge of the origins of the altered stiffness of the DYT1 dystonia patient-derived fibroblasts. For example, changes in nuclear physical properties and cell cycle stage can both lead to altered whole cell deformability (Kunda et al., 2008; Rowat et al., 2013; Swift et al., 2013; Otto et al., 2015).

While torsinA is required for the assembly of functional actin- and intermediate filament-binding LINC complexes (Nery et al., 2008; Saunders et al., 2017), it is interesting to note that DYT1 dystonia patient-derived fibroblasts exhibit increased nuclear strain following their exposure to mechanical stretch, suggesting that there are still physical forces pulling on the nucleus during stretch of the underlying substrate. The increased nuclear strain observed in fibroblasts isolated from DYT1 dystonia patients could be caused the nuclear envelope becoming more deformable due to the loss of torsinA function; the loss of functional LINC complexes could reduce the mechanical stability of the nuclear envelope by decoupling the inner nuclear membrane from the nuclear lamina and chromatin, which could lead to a larger increase in nuclear envelope area for a given magnitude of substrate stretch. The increased nuclear strain observed in DYT1 dystonia patient-derived fibroblasts may additionally be explained by the transmission of external forces to the nucleus via LINC complexes that associate with microtubules (Alam et al., 2014; Luxton and Starr, 2014; Chang et al., 2015b) and/or intermediate filaments (Hewett et al., 2006; Nery et al., 2014; Saunders et al., 2017). Physical forces could also be transmitted from the substrate to nucleus independently of LINC complexes. For example, nuclear pore complexes interact with the cytoplasmic microtubule motor proteins dynein and kinesins as well as nuclear lamins and chromatin (Yang et al., 2006; Splinter et al., 2010, 2012; Al-Haboubi et al., 2011; Steinberg et al., 2012). Future studies will test the relative contributions of specific components such as microtubules and nuclear pore complexes to the nuclear strain observed in mechanically stretched DYT1 dystonia patient-derived fibroblasts.

Given the role of torsinA and the LINC complex in regulating nuclear architecture, altered gene expression may be another potential mechanistic explanation for the altered cellular mechanotype we observed in human and mouse fibroblasts lacking torsinA function. While the expression of torsinA^Δ*E*^ in a cellular model of DYT1 dystonia was not sufficient to cause transcriptional dysregulation, a more recent unbiased transcriptomic analysis of embryonic brain tissue from *Tor1a*^+/Δ*E*^ and *Tor1a*^Δ*E*/Δ*E*^ mice revealed some changes in gene regulation (Beauvais et al., 2018). The role of altered gene expression in regulating the mechanotype of cells lacking torsinA function should thus be investigated. Furthermore, torsinA has been implicated in lipid metabolism (Grillet et al., 2016) and nuclear-cytoplasmic transport (Al-Haboubi et al., 2011; Chalfant et al., 2019), both of which could also contribute to cellular mechanotype.

We additionally discovered that the altered mechanotype of DYT1 patient-derived fibroblasts is associated with their decreased viability following mechanical stretch. These findings are consistent with previous reports that reduced levels of lamin A/C result in increased cell death following the migration of cells through narrow gaps (Harada et al., 2014). The nuclear rupture and DNA damage that results from mechanical stresses also depend on lamin A/C expression levels (Denais et al., 2016; Raab et al., 2016), substantiating that the mechanical stability of the nuclear envelope is critical for cell survival. The decreased viability of fibroblasts isolated from DYT1 dystonia patients could similarly result from an increased frequency of nuclear rupture and the accumulation of double stranded DNA breaks similar to previous reports in fibroblasts as well as cancer and immune cells (Denais et al., 2016; Raab et al., 2016). Alternatively, our findings of the decreased viability of DYT1 patient-derived fibroblasts following mechanical stretch might be explained by altered biochemical signaling triggered by mechanical stimuli that result in elevated levels of apoptosis (Jaalouk and Lammerding, 2009; Chan et al., 2011; Zhang et al., 2011). Future studies will define the mechanisms underlying the reduced survival of DYT1 dystonia patient-derived fibroblasts in response to mechanical stimuli.

It is tempting to speculate how altered cellular mechanotype may impact DYT1 dystonia pathogenesis. While our studies were conducted in fibroblasts and DYT1 dystonia is a neurological movement disorder (Fahn, 1988), molecular mediators of mechanotype are generally conserved across cell types (Rodriguez et al., 2003; Herrmann et al., 2007; Dittmer and Misteli, 2011; Chung et al., 2013; Chang et al., 2015b; Xavier et al., 2016; Fritz-Laylin et al., 2017). Thus, our findings of altered mechanotype in human and mouse fibroblasts may also be observed in neurons, which could have consequences for DYT1 dystonia pathogenesis. Since mechanoregulating proteins are often required for cellular functions that involve physical force generation, such as motility and mechanosensing (Anselme et al., 2018; Prahl and Odde, 2018), the impact of torsinA^Δ*E*^ on cellular mechanobiology could have deleterious consequences during tissue morphogenesis (Heisenberg and Bellaiche, 2013; Pizzolo et al., 2017). Indeed, previous studies showed that the migration of neurons in the dorsal forebrain of *Tor1a*^−/−^ mouse embryos *in vivo* (McCarthy et al., 2012) as well as the motility of torsinA-null MEFs and DYT1 dystonia patient-derived fibroblasts *in vitro* were impaired (Nery et al., 2008, 2014). Our observations of the reduced survival of DYT1 dystonia patient-derived fibroblasts following mechanical stretch incite further studies into the ability of neurons expressing torsinA^Δ*E*^ to sense the mechanical properties of their environment. Like all cells, neurons adapt their mechanotype by translating mechanical stimuli from their environment into biochemical signals through a process known as mechanotransduction (Franze et al., 2013). During development, the brain exhibits evolving stiffness gradients due to variations in the composition and architecture of its extracellular matrix (Franze et al., 2013; Barnes et al., 2017), which provide mechanical signals that instruct neuronal differentiation, proliferation, and survival (Iwashita et al., 2014; Koser et al., 2016). Thus, impaired mechanosensing of neurons in the developing brain may contribute to the manifestation of DYT1 dystonia.

More broadly, mutations in torsinA, LINC complex proteins, and their interacting partners have been implicated in numerous human diseases ranging from recessive neurological disorders associated with developmental delays to cardiomyopathy and muscular dystrophies (Luxton and Starr, 2014; Sewry and Goebel, 2014; Rebelo et al., 2015; Ghaoui et al., 2016; Janin et al., 2017; Kariminejad et al., 2017; Reichert et al., 2017). Further investigations of how torsinA and the LINC complex impact cellular mechanobiology could result in a deeper mechanistic understanding of human disease pathogenesis as well as the potential discovery of novel therapeutic targets.

## Author Contributions

NG, AR, and GL designed the research. NG performed all the experiments and analyzed the data. CL performed the q-DC experiments. PK and NG conducted the cell stretching experiments. CS generated the lentiviral transduced stable NIH3T3 fibroblast lines. LF and SY generated the *LMNA* and *LMNB1* MEFs. NG, AR, and GL wrote the manuscript. All authors reviewed the manuscript.

## Conflict of Interest Statement

The authors declare that the research was conducted in the absence of any commercial or financial relationships that could be construed as a potential conflict of interest.

## Footnotes

1 https://github.com/knybe/RowatLab-DC-Analysis

## Abbreviations

AAA+: ATPase-associated with various cellular activities
*d*: cell diameter
*E*_a_: apparent cell elastic modulus
*C*: circularity
LAP1: lamina-associated polypeptide 1
LULL1: luminal domain-like LAP1
LINC: linkers of nucleoskeleton and cytoskeleton
MEF: mouse embryonic fibroblast
Nesprin: Nuclear envelope spectrin repeat
NS: Not significant
PDMS: Polydimethylsiloxane
PMF: parallel microfiltration
q-DC: quantitative deformability cytometry
SUN: Sad1/UNC-84
TAN: transmembrane actin-associated nuclear
*TT*: transit time
WT: wild type.
