# Supplementary material for: DYT1 Dystonia Patient-Derived Fibroblasts Have Increased Deformability and Susceptibility to Damage by Mechanical Forces
Source: Front Cell Dev Biol. 2019 Jun 26;7:103. doi: 10.3389/fcell.2019.00103 (PMC6606767; doi:10.3389/fcell.2019.00103)
Supplement: Supplementary file 1 [file Data_Sheet_1.PDF]

Supplementary Material

Supplementary Material

DYT1 dystonia patient-derived fibroblasts have increased deformability and susceptibility to damage by mechanical forces

Navjot Kaur Gill, Chau Ly, Paul H. Kim, Cosmo A. Saunders, Loren G. Fong, Stephen G. Young, G.W. Gant Luxton*, Amy C. Rowat*

*** Correspondence:** G.W. Gant Luxton: gwgl@umn.edu; Amy C. Rowat: rowat@ucla.edu

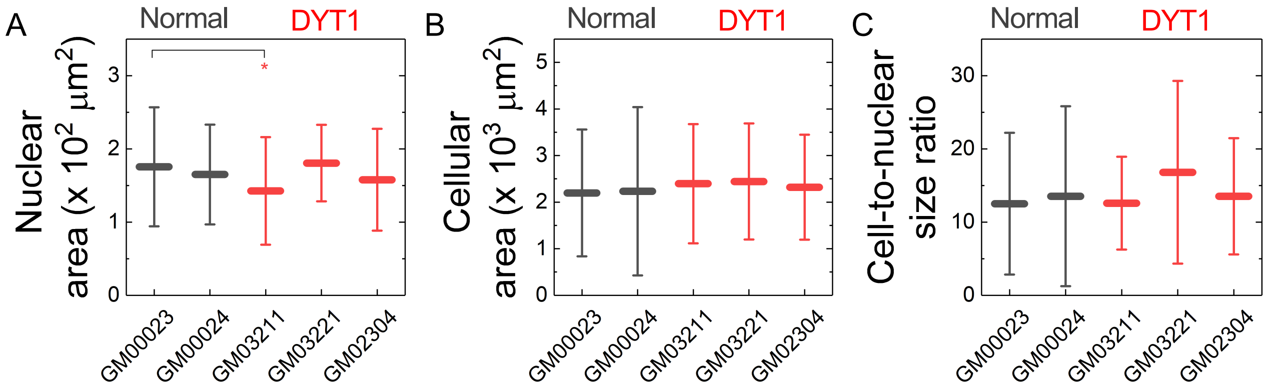

Supplementary Figure 1. Quantification of cell and nuclear area of normal and DYT1 dystonia patient-derived fibroblasts. (**A**) Nuclear area, (**B**) cellular area, and (**C**) cell-to-nucleus area ratio for the indicated fibroblasts measured while adhered to glass coverslips. Each data point represents the mean ± SD. Data were obtained from three independent experiments. Statistical significance was determined using the Mann-Whitney U test and indicated where significant. * $p < 0.05$. Not significant (NS) $p > 0.05$ is not indicated on these plots for clarity.

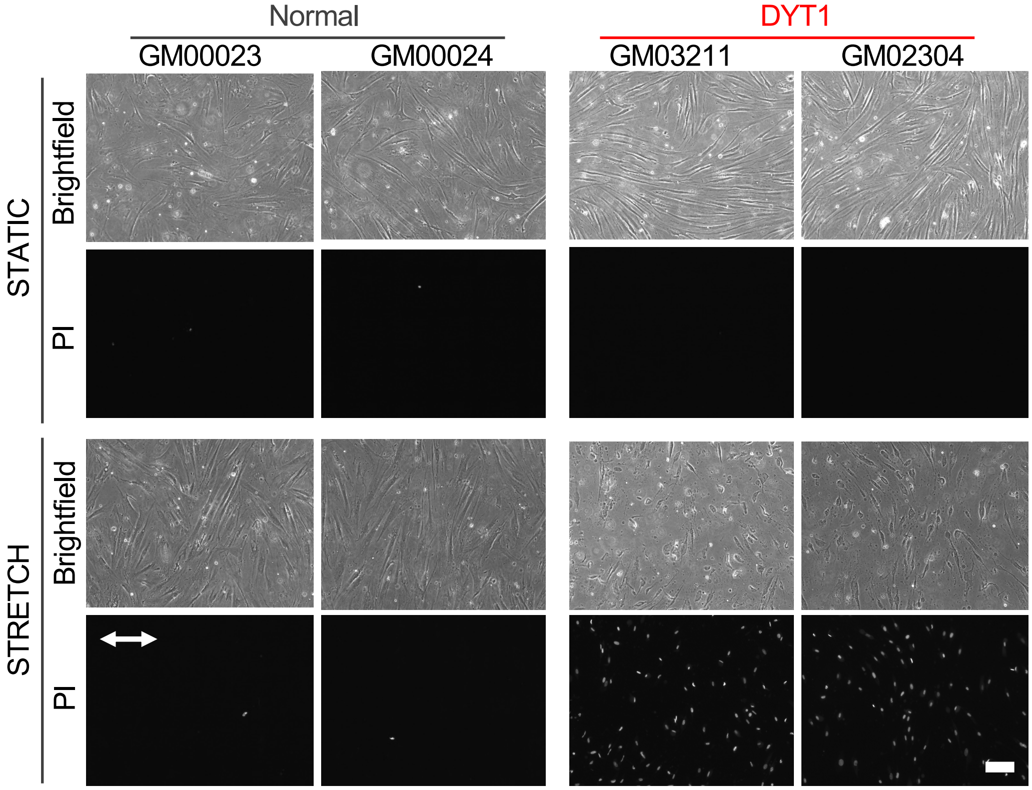

Supplementary Figure 2. DYT1 dystonia patient-derived fibroblasts have reduced viability after mechanical stretching compared to normal fibroblasts. Representative brightfield and epifluorescence images of propidium iodide (PI)-labeled cells after stretching (stretch) compared to cells that were not exposed to mechanical stretching (static). PI uptake indicates cell death. White arrows show the direction of uniaxial stretch applied to the substrate. Scale, 100 μm.

CV (%)	GM00023	GM00024	GM03211	GM02304
**Replicate 1**	27.6	26.8	24.7	28.3
**Replicate 2**	26.4	21.9	25.0	20.0

Supplementary Table 1. Variability in nuclear area across samples and replicates. Coefficient of variation (CV) is determined for nuclear area of normal and DYT1 dystonia patient-derived fibroblasts plated on PDMS substrates. Using the Levene's test, we determined that variance in nuclear shape was statistically similar across all samples and replicates ($p = 1$ for all pairwise comparisons).
